# PCOS without hyperandrogenism is associated with higher plasma Trimethylamine N-oxide levels

**DOI:** 10.1186/s12902-019-0486-9

**Published:** 2020-01-06

**Authors:** Jiayu Huang, Lin Liu, Chunyan Chen, Ying Gao

**Affiliations:** Department of Gynecology and Obstetrics, Union Hospital, Tongji Medical College, Huazhong University of Science and Technology, Wuhan, China

**Keywords:** PCOS, Trimethylamine N-oxide, Inflammation, Insulin resistance

## Abstract

**Background:**

Polycystic ovary syndrome (PCOS) is an endocrine and metabolic disorder, and its pathogenesis is still under debate. Trimethylamine-N-oxide (TMAO) is a small, organic compound generated by the gut microbiome with a hypothesized relation to insulin resistance (IR) and low-grade inflammation in PCOS. By comparing plasma TMAO levels in non-PCOS participants and PCOS patients without hyperandrogenism (HA), we aimed to determine whether plasma TMAO levels correlate with PCOS without HA and to analyze their relationship with low-grade inflammation and IR.

**Methods:**

A total of 27 PCOS patients without HA and 23 non-PCOS participants were enrolled in this study and subdivided into “nonobese” and “obese” arms for each group. Levels of plasma TMAO were quantified, and basic clinical characteristics and plasma biomarkers of inflammation were assessed.

**Results:**

First, plasma TMAO levels, insulin levels and homeostatic model assessment of insulin resistance (HOMA-IR) values were higher in PCOS patients without HA, especially in the obese subgroup. Second, the levels of the inflammatory factors interleukin (IL)-17A, IL-18 and interferon gamma (IFN-γ) were significantly increased in obese PCOS patients without HA. Third, plasma TMAO levels were associated with body mass index (BMI) in the normal-weight groups, and the obese groups had higher fasting plasma insulin (FINS) and HOMA-IR values. Finally, logistic regression showed that the plasma levels of TMAO and luteinizing hormone/follicle-stimulating hormone (LH/FSH) were independent predictors of PCOS and indicated an increased risk of PCOS.

**Conclusions:**

Elevated plasma TMAO levels may be associated with the pathogenesis of PCOS without HA and correlated with increased systemic inflammation. Further studies are needed to determine the suitability of TMAO as a predictive biomarker and to identify possible therapies for PCOS.

## Background

Polycystic ovary syndrome (PCOS) is an endocrine and metabolic disorder commonly found in women of childbearing age. Common clinical criteria include menstrual disorder, infrequent ovulation, chronic anovulation, hirsutism, hyperandrogenism (HA), insulin resistance (IR) and ovarian polycystic changes [1, 2]. Currently, the mechanism of PCOS is still under debate. However, it is acknowledged that low-grade inflammation and IR are involved in the pathological process of PCOS [1, 3, 4].

PCOS is usually diagnosed according to the Rotterdam criteria, given the presence of at least two of three criteria, clinical or laboratory HA, ovulatory dysfunction or polycystic ovaries (PCO) on ultrasound [5]. The Rotterdam criteria divide PCOS into different phenotypes: hyperandrogenic anovulatory women with PCO on ultrasound (phenotype A); hyperandrogenic anovulatory women without PCO (phenotype B); hyperandrogenic ovulatory women with PCO (phenotype C) or nonhyperandrogenic anovulatory women with PCO (phenotype D) [6]. Different types of PCOS have different manifestations and metabolic features, which significantly increase the risk of long-term cardiovascular disease (CVD), diabetes and tumors, seriously threaten patient physical and mental health, and may even pass to the next generation [7]. The prevalence of the four phenotypes varies in different ethnic groups, whereas in China, nonhyperandrogenic anovulatory women with PCO (phenotype D) account for a considerable proportion of the women with anovulatory infertility problems [8]. Thus, investigating the pathogenesis of non-HA PCOS is of great significance. The majority of the literature on PCOS to date has focused on the diagnosis of HA, and research on the metabolic implications of the non-HA phenotype is relatively limited.

The intestinal flora is considered to be “the second brain” or “the second genome” of human beings and affects crucial physiological functions such as metabolism, immunity and signaling pathways of the host through long-term low-level exposure to metabolic and decay products [9]. Damaged metabolic health is associated with relatively low microbiota gene expression levels and decreased microbial diversity [10]. New studies have shown that the occurrence of PCOS is associated with changes in the intestinal flora [11–13]. The changes in PCOS-related intestinal flora and its metabolites may provide new mechanistic insights into the pathogenesis and targets for the treatment of PCOS.

Trimethylamine N-oxide (TMAO) is a small, organic, gut microbiome-generated compound [14]. Dietary phosphatidylcholine, choline, and carnitine are metabolized to trimethylamine (TMA) under the action of certain intestinal microbes. After absorption into the blood, TMA is carried via the portal circulation to the liver, where flavin-containing monooxygenase (FMO) plays a major role in rapidly converting it into TMAO [15]. Studies have shown that TMAO upregulates scavenger receptors in macrophages and promotes the accumulation of cholesterol in macrophages and the formation of foam cells, thereby promoting the formation of vascular plaques and inflammatory responses via the MAPK and NF-κB pathways [16]. TMAO also enhances platelet hyperreactivity and thrombosis risk and has adverse effects on cardiovascular function [17, 18]. Type II diabetes and increased age and body mass index (BMI) are associated with increased concentrations of TMAO in plasma [19, 20]. Studies have also suggested that elevated levels of plasma insulin caused by IR increase FMO3 activity and further increase serum levels of TMAO [21]. Since TMAO plays a role in IR or inflammation in CVD, type II diabetes and thrombosis, is it related to IR or low-grade inflammation in PCOS?

In this transversal study, we aimed to detect low-grade inflammation in PCOS without HA, determine whether plasma TMAO levels are correlated with PCOS without HA and analyze its relationship with low-grade inflammation and IR.

## Methods

### Patients and study design

All patients were recruited from the In Vitro Fertilization (IVF) Center of Wuhan Union Hospital, Tongji Medical College, Huazhong University of Science and Technology between January 2019 and July 2019. The 27 patients diagnosed with PCOS without HA by the two other Rotterdam criteria, oligoanovulation (OA), PCO on ultrasound, blood total testosterone levels < 2.6 nM and no clinical HA (Ferriman–Gallwey score < 8) were included in the PCOS-N group [22]. In contrast, 23 women with tubal factor infertility or those resorting to IVF assistance because of male infertility were included as the control group. The exclusion criteria for the study groups included the use of hormonal birth control methods, including the specific use of oral contraceptives, fertility medications, metformin or antibiotics during the prior 3 months. We divided the participants into 4 subgroups based on BMI > 24 according to the “Guide1ines for Prevention and Control of Overweight and Obese Adults in China” because TMAO has been associated with BMI [20]: the normal-weight PCOS-N group, obese PCOS-N group, normal-weight control group and obese control group [23]. This study was approved by the Institutional Review Board (S046) of Wuhan Union Hospital, Tongji Medical College, HUST, and all patients gave written informed consent before participation. The whole study adhered to STROBE guidelines/methodology.

### Detection of baseline characteristics

Blood samples were collected from participants at an early follicular phase (days 3–5) of menstruation and analyzed with standard laboratory techniques by an automatic biochemical analyzer at Wuhan Union Hospital. Blood samples were collected after a 12 h overnight fast to detect luteinizing hormone (LH), follicle-stimulating hormone (FSH), progesterone (P), estradiol (E2), testosterone (T) and fasting plasma insulin (FINS) by using radioimmunoassay; creatinine (Cr) was detected by SOP-cr; free testosterone (FT) was detected by ChemiLuminescence; triglyceride (TG) was detected by GPO-PAP; cholesterol (CHOL) was detected by CHOD-PAP; alanine transaminase (ALT) and aspartate aminotransferase (AST) were detected by rate method; high-density lipoprotein (LDL), high-density lipoprotein (HDL) and low-density small dense LDL (sdLDL) were measured by enzymic method; turbidimetric inhibition immuno assay was used to detect C-reactive protein (CRP); apolipoprotein A (APOA), apolipoprotein B (APOB); hexokinase method was used to detect fasting blood glucose (FBG) to calculate HOMA-IR.

### Analysis of TMAO and inflammatory factors in plasma

Stable isotope dilution liquid chromatography-tandem mass spectrometry (LC-MS/MS) was used for the quantification of plasma TMAO (ACQUITY UPLC H- Class/Xevo G2 TQ-XS MS/MS) by Metabo-Profile, Shanghai, P. R. China. Inflammatory factors including interferon alpha (IFN-α), interferon gamma (IFN-γ), tumor necrosis factor alpha (TNF-α), monocyte chemoattractant protein-1 (MCP-1), interleukin 6 (IL-6), interleukin 8 (IL-8), interleukin 10 (IL-10), interleukin 12p70 (IL-12p70), interleukin 17A (IL-17A), interleukin 18 (IL-18), and interleukin 33 (IL-33) were detected by flow cytometry (Celesta, BD) using a Human Inflammation Panel 1 kit (LEGENDplex™).

### Statistical analysis

Data are non-normally distributed and expressed as the median (interquartile range). Clinical characteristics were compared by nonparametric tests. All statistical tests were 2-tailed. A *P*-value < 0.05 was considered statistically significant. Statistical analysis was performed using STATA version 12.0 (StataCorp) and SPSS version 22.0 (IBM).

## Results

### Baseline characteristics

There is no participant with missing data in the whole study. The baseline characteristics summarized the clinical and laboratory characteristics with *P* values for the comparison of PCOS without HA and non-PCOS in this study. No significant differences were observed in age among the groups (*P* > 0.05). As expected, the levels of LH, LH/FSH, T, and FBG and HOMA-IR values in women in the PCOS-N group were significantly higher than those in the control group (Table 1). In the comparison of subgroups fasting plasma insulin levels and HOMA-IR were higher in the obese groups, and CRP and ALT levels were found to be increased in the PCOS-obese group. Additionally, AST levels were increased in the obese control group (Table 2).

**Table 1:** Baseline characteristics of non-PCOS and PCOS without HA participants

	Control	PCOS-N	*P*-Value
n	23	27	
Age (years)	27.00 (24.00–29.00)	27.00 (25.00–29.00)	0.746
BMI (kg/m^2^)	24.77 (21.23–27.48)	24.46 (21.09–28.60)	0.853
LH (IU/L)	5.35 (4.21–7.01)	9.80 (7.06–16.57)	0.001*
FSH (IU/L)	6.33 (5.87–7.78)	5.77 (3.22–7.34)	0.056
LH/FSH	0.83 (0.67–1.25)	1.77 (1.35–2.77)	0.0001**
P (nmol/L)	0.63 (0.36–0.99)	0.57 (0.41–1.05)	0.619
E2 (pmol/L)	149.30 (113.60–204.30)	189.70 (114.70–274.60)	0.490
T (nmol/L)	1.10 (0.75–1.50)	1.22 (1.08–1.44)	0.164
FT (pmol/L)	7.18 (6.47–10.03)	8.17 (6.01–11.39)	0.697
FBG (mmol/L)	5.60 (5.20–6.00)	6.00 (5.60–6.50)	0.009*
FINS (μIU/ml)	11.90 (8.91–17.02)	14.62 (11.00–19.62)	0.158
HOMA-IR	2.82 (2.16–4.35)	3.65 (2.98–5.58)	0.044
Cr (μmol/L)	64.40 (59.00–66.50)	63.90 (59.10–67.30)	0.884
CRP (mg/L)	1.16 (0.78–2.63)	1.52 (0.82–3.40)	0.683
CHOL (mmol/L)	4.80 (4.27–5.13)	4.80 (4.21–5.61)	0.633
TG (mmol/L)	1.03 (0.86–1.49)	1.39 (0.74–2.02)	0.640
ALT (U/L)	19.00 (11.00–27.00)	21.00 (13.00–32.00)	0.465
AST (U/L)	17.00 (16.00–22.00)	19.00 (16.00–22.00)	0.464
HDL (mmol/L)	1.42 (1.27–1.59)	1.32 (1.20–1.64)	0.823
LDL (mmol/L)	2.72 (2.28–3.49)	2.60 (2.00–3.21)	0.533
sdLDL (mmol/L)	0.87 (0.71–1.06)	0.95 (0.67–1.34)	0.546
APOA (g/L)	1.35 (1.25–1.64)	1.40 (1.30–1.53)	0.533
APOB (g/L)	0.73 (0.63–0.80)	0.69 (0.63–0.83)	0.853

Data are presented as median (interquartile range). (*) represents significant difference between groups, *p* < 0.01; (**) represents significant difference between groups, *p* < 0.0001. *BMI* Body mass index, *LH* luteinizing hormone, *FSH* Follicle-stimulating hormone, *P* Progesterone, *E2* Estradiol, *T* Testosterone, *FT* Free testosterone, *FBG* Fasting blood glucose, *FINS* Fasting plasma insulin, *HOMA-IR* Homeostasis model assessment insulin resistance index, *Cr* Creatinine, *CRP* C-reactive protein, *CHOL* Cholesterol, *TG* Triglyceride, *ALT* Alanine transaminase, *AST* Aspartate aminotransferase, *LDL* Low-density lipoprotein, *HDL* High-density lipoprotein, *sdLDL* Small dense LDL, *APOA* Apolipoprotein A, *APOB* Apolipoprotein B

**Table 2:** Baseline characteristics of subgroups of non-PCOS and PCOS without HA participants

	Control		PCOS-N	
	BMI < 24	BMI ≥ 24	BMI < 24	BMI ≥ 24
n	11	12	11	16
Age (years)	27.00 (26.00–29.00)	26.50 (22.5–28.75)	26.00 (24.00–29.00)	27.00 (25.50–29.75)
BMI (kg/m^2^)	21.23 (19.35–22.20)	27.39 (25.99–28.90)^a^	20.83 (19.20–22.58)^b^	27.19 (24.65–29.59) ^ac^
LH (IU/L)	5.21 (4.21–5.63)	6.16 (5.00–11.13)	8.72 (6.67–15.23)	11.33 (7.26–20.24)^a^
FSH (IU/L)	6.96 (5.32–9.36)	6.28 (5.97–7.41)	5.43 (3.20–8.51)	5.82 (3.60–7.17)
LH/FSH	0.79 (0.60–0.91)	1.01 (0.82–1.54)	1.62 (1.33–2.42)^a^	1.90 (1.36–2.87)^ab^
P (nmol/L)	0.70 (0.60–0.91)	0.52 (0.37–0.98)	0.77 (0.45–1.75)	0.51 (0.38–0.88)
E2 (pmol/L)	130.80 (94.00–175.40)	168.65 (132.85–381.08)	230.00 (120.40–585.30)	159.70 (108.4–235.35)
T (nmol/L)	1.10 (0.61–1.37)	0.90 (0.76–1.59)	1.37 (1.09–1.56)	1.18 (0.95–1.40)
FT (pmol/L)	7.42 (5.95–9.93)	6.92 (6.51–10.11)	6.01 (4.93–10.45)	9.99 (6.71–13.34)
FBG (mmol/L)	5.60 (5.20–6.00)	5.70 (5.05–5.98)	5.80 (5.60–6.30)^b^	6.10 (5.70–6.58)
FINS (μIU/ml)	8.99 (7.40–10.80)	16.88 (12.53–20.51)^a^	12.78 (8.98–14.65)	15.79 (11.95–30.87)^a^
HOMA-IR	2.38 (1.91–2.59)	3.98 (3.00–5.22)^a^	3.57 (2.27–3.65)	4.16 (3.15–8.99)^a^
Cr (μmol/L)	64.40 (59.00–66.20)	64.30 (61.08–66.50)	59.70 (56.20–69.00)	64.95 (63.50–67.28)
CRP (mg/L)	1.02 (0.46–2.61)	1.56 (1.06–3.16)	0.84 (0.20–1.37)	2.15 (1.53–5.58)^ac^
CHOL (mmol/L)	4.80 (4.01–5.34)	4.75 (4.31–5.11)	4.41 (3.96–4.81)	5.11 (4.69–6.01)
TG (mmol/L)	1.03 (0.90–1.24)	1.01 (0.80–1.86)	0.81 (0.60–1.43)	1.53 (1.13–2.24)
ALT (U/L)	13.00 (8.00–19.00)	24.00 (18.25–35.00)	15.00 (10.00–21.00)	28.00 (15.75–36.25)^ac^
AST (U/L)	16.00 (13.00–17.00)	20.00 (16.25–28.00)^a^	17.00 (15.00–22.00)	20.00 (16.50–21.75)
HDL (mmol/L)	1.42 (1.27–1.59)	1.40 (1.23–1.59)	1.63 (1.31–1.85)	1.25 (1.13–1.51)
LDL (mmol/L)	2.72 (2.28–3.49)	2.77 (2.26–3.38)	2.44 (1.90–3.01)	2.85 (2.52–3.39)
sdLDL (mmol/L)	0.87 (0.73–1.06)	0.84 (0.60–1.17)	0.77 (0.57–0.95)	1.18 (0.76–1.54)
APOA (g/L)	1.40 (1.21–1.64)	1.35 (1.26–1.63)	1.46 (1.33–1.57)	1.38 (1.30–1.53)
APOB (g/L)	0.73 (0.64–0.80)	0.72 (0.62–0.83)	0.65 (0.54–0.71)	0.78 (0.64–0.87)

Data are presented as median (interquartile range). ^a^*P* < 0.05 versus control-normal weight participants, ^b^*P* < 0.05 versus control-obese weight participants, ^c^*P* < 0.05 versus PCOS-normal weight participants. *BMI* Body mass index, *LH* Luteinizing hormone, *FSH* Follicle-stimulating hormone, *P* Progesterone, *E2* Estradiol, *T* Testosterone, *FT* Free testosterone, *FBG* Fasting blood glucose, *FINS* Fasting plasma insulin, *HOMA-IR* Homeostasis model assessment insulin resistance index, *Cr* Creatinine, *CRP* C-reactive protein, *CHOL* Cholesterol, *TG* Triglyceride, *ALT* Alanine transaminase, *AST* Aspartate aminotransferase, *LDL* Low-density lipoprotein, *HDL* High-density lipoprotein, *sdLDL* Small dense LDL, *APOA* Apolipoprotein A, *APOB* apolipoprotein B

### Increased plasma levels of TMAO and inflammatory factors

**Fig. 1:**
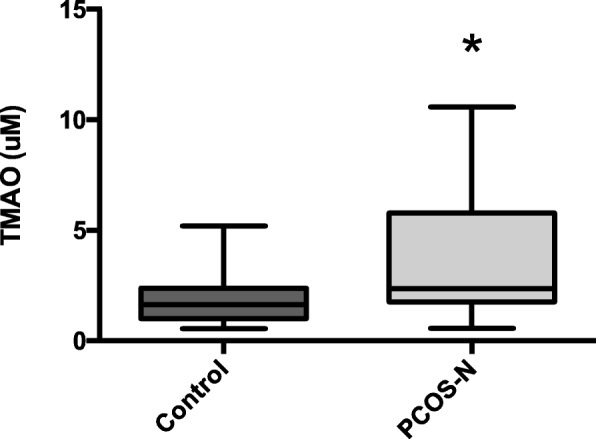
Plasma trimethylamine N-oxide (TMAO) levels in control group and polycystic ovary syndrome (PCOS) without hyperandrogenism (HA) group. Data presented using box-and-whisker plot with central line representing median, box representing interquartile range and bars representing highest and lowest value. (*) represents significant difference between groups, *p* < 0.01)

The overall plasma levels of TMAO were significantly elevated in PCOS without HA patients compared with the non-PCOS patients (Table 3) (Fig. 1). Moreover, in the subgroups, serum IL-18 and plasma TMAO levels were increased in the obese PCOS-N group; serum IL-17A and IFN-γ levels were higher in obese people and IL-17A might be worsened by PCOS (Table 4).

**Table 3:** Baseline characteristics of non-PCOS and PCOS without HA participants

	Control	PCOS-N	*P*-Value
IFN-α (pg/ml)	3.02 (2.53–4.61)	3.11 (1.94–4.15)	0.763
IFN-γ (pg/ml)	4.16 (3.03–5.70)	4.52 (3.61–5.99)	0.477
TNF-α (pg/ml)	3.34 (2.27–3.44)	3.24 (2.19–3.54)	0.646
MCP-1 (pg/ml)	154.38 (94.27–218.21)	167.53 (130.18–197.62)	0.613
IL-6 (pg/ml)	3.78 (3.66–7.90)	3.93 (3.66–9.23)	0.362
IL-8 (pg/ml)	11.47 (9.01–16.01)	11.03 (9.30–14.41)	0.603
IL-10 (pg/ml)	5.99 (3.72–7.66)	4.69 (3.76–8.07)	0.837
IL-17A (pg/ml)	1.89 (1.08–1.94)	1.92 (1.34–2.29)	0.179
IL-18 (pg/ml)	272.61 (177.57–377.55)	310.25 (209.55–490.93)	0.521
IL-33 (pg/ml)	37.71 (18.84–45.74)	31.60 (12.77–49.29)	0.785
Plasma TMAO (μmol/L)	1.65 (1.01–2.38)	2.37 (1.77–5.78)	0.003*

Data are presented as median (interquartile range). (*) represents significant difference between groups, *p* < 0.01; (**) represents significant difference between groups, *p* < 0.0001. *IFN-α* Interferon alpha, *IFN-γ* Interferon gamma, *TNF-α* Tumor necrosis factor alpha, *MCP-1* Monocyte chemoattractant protein-1, *IL-6* Interleukin 6, *IL-8* Interleukin 8, *IL-10* Interleukin 10, *IL-17A* Interleukin 17A, *IL-18* Interleukin 18, *IL-33* Interleukin 33, *TMAO* Trimethylamine N-oxide

**Table 4:** Plasma concentrations of inflammatory factors and TMAO in subgroups of non-PCOS and PCOS without HA cases

	Control		PCOS-N	
	BMI < 24	BMI ≥ 24	BMI < 24	BMI ≥ 24
IFN-α (pg/ml)	2.61 (2.01–3.11)	3.91 (2.75–4.70)	3.04 (2.78–4.15)	3.54 (1.88–4.44)
IFN-γ (pg/ml)	2.73 (2.27–3.44)	5.38 (4.16–6.50)^a^	3.97 (3.21–5.14)	5.10 (4.16–6.48)^a^
TNF-α (pg/ml)	2.73 (2.27–3.24)	3.44 (2.51–3.67)	3.17 (2.32–3.44)	3.04 (2.78–4.15)
MCP-1 (pg/ml)	118.44 (93.42–231.53)	157.41 (122.61–211.24)	149.68 (126.77–196.24)	167.63 (130.55–223.05)
IL-6 (pg/ml)	3.78 (3.66–9.00)	3.78 (3.66–6.46)	3.78 (3.66–7.22)	5.18 (3.66–15.09)
IL-8 (pg/ml)	10.40 (9.01–16.01)	11.83 (9.03–15.76)	9.30 (9.00–12.64)	11.27 (9.30–18.57)
IL-10 (pg/ml)	6.65 (3.72–7.01)	5.48 (3.72–7.87)	5.31 (3.72–7.66)	4.67 (3.84–8.61)
IL-17A (pg/ml)	1.30 (0.94–1.64)	1.94 (1.90–2.24)^a^	1.90 (1.03–2.01)	2.25 (1.89–2.82)^ac^
IL-18 (pg/ml)	255.89 (175.33–351.69)	354.17 (272.48–431.46)	209.55 (174.31–272.67)	405.12 (299.34–546.30)^ac^
IL-33 (pg/ml)	37.71 (20.57–74.61)	36.79 (18.86–45.30)	37.99 (24.70–43.98)	30.18 (7.36–67.58)
Plasma TMAO (μmol/L)	1.65 (0.75–2.09)	1.64 (1.15–2.59)	2.18 (1.77–3.86)	3.01 (1.59–7.17)^a^

Data are presented as median (interquartile range). ^a^*P* < 0.05 versus control-normal weight participants, ^b^*P* < 0.05 versus control-obese weight participants, ^c^*P* < 0.05 versus PCOS-normal weight participants. *IFN-α* Interferon alpha, *IFN-γ* Interferon gamma, *TNF-α* Tumor necrosis factor alpha, *MCP-1* Monocyte chemoattractant protein-1, *IL-6* Interleukin 6, *IL-8* Interleukin 8, *IL-10* Interleukin 10, *IL-17A* Interleukin 17A, *IL-18* Interleukin 18, *IL-33* Interleukin 33, *TMAO* Trimethylamine N-oxide

### Associations between plasma levels of TMAO and PCOS without HA as well as biomarkers of inflammation

A partial correlation was calculated to analyze whether TMAO correlated with PCOS without HA by controlling for the confounding factor BMI, and the results showed that the plasma levels of TMAO (*r* = 0.423, *P* < 0.01), LH (*r* = 0.482, *P* < 0.01), LH/FSH (*r* = 0.460, *P* < 0.01), and FBG (*r* = 0.408, *P* < 0.01) were positively correlated with the incidence of PCOS without HA. Logistic regression was utilized to examine the biomarker in relation to the odds of PCOS without HA compared to controls. Use statistically significant variables in partial correlation analysis as independent variables in logistic regression. The adjusted OR proved a statistically significant association between plasma levels of TMAO (OR = 3.814; 95% CI: 1.330–10.939, *P*-value = 0. 013), LH/FSH (OR = 18.008; 95% CI: 1.012–320.449, *P*-value = 0.049) and PCOS (Table 5). Females with higher plasma TMAO level and LH/FSH ratio are 3.8 and 18.0 times more likely to present PCOS, respectively.

**Table 5:** Logistic regression analysis was used to examine the relationship of variable levels in two groups (PCOS-N and Control)

Model		β	Sig	Exp (B)	95% C.I. for EXP (B)	
					Lower	Upper
	TMAO	1.339	0.013	3.814	1.330	10.939
	BMI	−0.205	0.279	0.814	0.561	1.182
	LH	0.281	0.366	1.324	0.720	2.437
	LH/FSH	2.891	0.049	18.008	1.012	320.449
	FBG	4.733	0.053	113.663	0.946	13,643.650
	Constant	−31.839	0.029	0		

*TMAO* Trimethylamine N-oxide, *BMI* Body mass index, *LH* Luteinizing hormone, *FSH* Follicle-stimulating hormone, *FBG* Fasting blood glucose

**Fig. 2:**
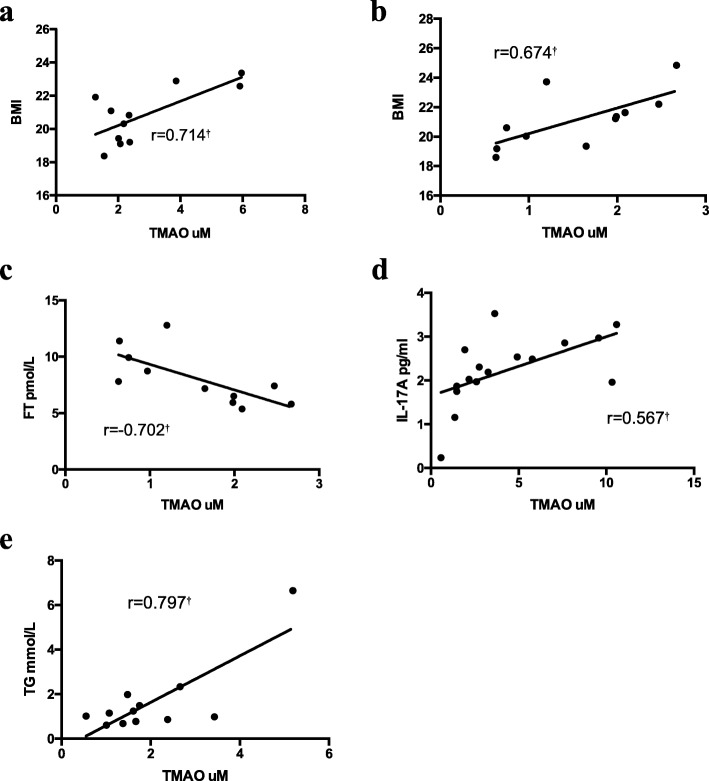
**a** Correlations between plasma trimethylamine N-oxide (TMAO) and BMI in normal-weight PCOS-N group; **b** Correlations between plasma TMAO and BMI in normal-weight control group; **c** Correlations between plasma TMAO and FT in normal-weight control group; **d** Correlations between plasma TMAO and IL-17A in obese PCOS-N group; **e** Correlations between plasma TMAO and TG in obese control group. Spearman correlations performed and presented for each group. (†) represents significant correlation (*p* < 0.05). BMI, Body mass index; FT, free testosterone; IL-17A, interleukin 17A; TG, triglyceride

In the normal-weight PCOS-N groups, the plasma TMAO levels were positively correlated with BMI (*r* = 0.714, *P* < 0.05). In the normal-weight control group, the plasma TMAO levels were also positively correlated with BMI (*r* = 0.674, *P* < 0.05). and negatively correlated with FT (*r* = − 0.702, *P* < 0.05). Moreover, in the obese PCOS-N group, the plasma levels of TMAO were positively correlated with IL-17A (*r* = 0.567, *P* < 0.05), whereas in obese control group, the plasma levels of TMAO were positively correlated with TG (*r* = 0.797, *P* < 0.05). (Fig. 2).

## Discussion

In this study, we tested the hypothesis that plasma concentrations of the gut microbe-generated metabolite TMAO were associated with PCOS patients without HA. Several key findings were noted in this analysis. First, plasma TMAO levels and HOMA-IR were higher in PCOS patients without HA, especially in obese patients. Second, though part of the overall mean value of inflammatory factor levels in participants with PCOS without HA was higher than that of the control group, the personal inflammatory factor levels varied among patients, and only inflammatory factor IL-17A, IL-18 and IFN-γ levels were found to be significantly increased in obese PCOS patients without HA. Moreover, the plasma levels of TMAO were significantly and positively correlated with IL-17A in the obese PCOS-N group. Third, plasma TMAO levels were associated with BMI in the normal-weight groups, and the obese groups had higher FINS and HOMA-IR values. Finally, logistic regression showed that plasma levels of TMAO and LH/FSH were both independent predictors of PCOS and indicated an increased risk of PCOS. That is, a higher plasma TMAO level or LH/FSH ratio increases the risk of PCOS.

Several mechanisms may be involved in the association between increased plasma levels of TMAO and clinical manifestation of PCOS without HA. Previous studies support our findings that CRP levels and serum IL-18 were higher in PCOS patients [24–26]. IL-18 is a member of the interleukin-1 (IL-1) family, which is mainly produced by immune cells such as monocytes, NK cells and macrophages and mediates the inflammatory response [27]. IL-18 levels were increased in PCOS patients and correlated with IR and obesity [28]. Our research shows that IL-17A plays an important role in TMAO levels in obese PCOS patients without HA and might lose its relative importance in modulating TMAO levels in normal-weight PCOS patients. This finding does not mean that IL-17A has no role in PCOS but rather that is has a weaker correlation with TMAO in the context of PCOS. IL-17A has been shown to be involved in the pathogenesis of lipid metabolism and atherosclerosis [29], suggesting that IL-17A has a role in the pathogenesis of PCOS [30]. Chronic inflammation affects follicular development in PCOS and is closely related to pathological changes such as IR, obesity and hyperactivity.

Liu, R. reported a significant structural shift of the gut microbiota (represented by a reduction in alpha diversity, an increase in LPS-producing bacteria, and a decrease in spore-forming species) in patients with PCOS; gut microbial dysbiosis in both obese and nonobese women shares similar composition with that of obesity and is associated with the phenotypes of PCOS [13]. Many other toxins or substances produced by abnormal gut microbiota are known to cause metabolic disruptions, among which many are present in PCOS, including lipopolysaccharide (LPS). A positive correlation was observed between TMAO and LPS in T2DM-CKD subjects (*r* = 0.456, *p* = 0.04, [31]). It was demonstrated that in PCOS, lipid-induced LPS-mediated inflammation through TLR-4 was associated with obesity and worsened by PCOS [32]. The above studies are in line with our finding that plasma TMAO levels were higher in the obese PCOS-N group. An increasing number of studies have shown that inflammation, disordered lipid metabolism, and intestinal flora interact with each other to affect the internal environment of the organism. PCOS is a sophisticated complex in which inflammation, disordered lipid metabolism, and intestinal flora contribute differently in different patients. The change in intestinal flora may also cause changes in plasma TMAO levels.

The chronic low-grade inflammatory state of PCOS is closely associated with the occurrence of IR. According to previous studies, the level of most low-grade chronic inflammation markers, such as TNF-α, CRP, and IL-6, is positively correlated with IR and circulating insulin levels [33]. Inflammatory factors stimulate serine, leading to increased levels of phosphorylation, inhibited tyrosine kinase activity, and decreased levels of insulin-stimulated receptor tyrosine phosphorylation, impeding insulin receptor signaling, and reducing IRS-1 activity. Decreased expression of GLUT-4 leads to impaired glucose uptake.

In our research, though obese and PCOS-N participants were likely to have a higher HOMA-IR, we did not find a significant positive correlation between plasma TMAO and IR. Some studies demonstrate a potential mechanism linking TMAO to IR in which TMAO-dependent increased levels of N-Nitroso Compounds (NOCs) have been shown to be a driver of IR diseases [34]. Another proposed mechanism between IR and TMAO levels is that IR and the corresponding increased plasma levels of insulin upregulate the activity of flavin-containing monooxygenase 3 (FMO-3) and hence increase TMAO levels [35], indicating that high TMAO levels might contribute to the onset of PCOS. The research shows obese people were likely to have higher average level of FINS, CRP and ALT, worsened by PCOS. Also, these elevated markers indicated the exacerbation of the additional risk for women with PCOS when they gain weight, compared to the risk of weight gain among women without PCOS. Serum CRP levels increased even more in obese PCOS women, indicating that women with PCOS should be exceedingly careful to avoid weight gain due to their exacerbated risks. Policies for the prevention of obesity should be even stronger for women with PCOS.

To the best of our knowledge, this is the first study to report plasma TMAO levels in PCOS patients without HA compared with non-PCOS and to investigate whether plasma TMAO levels are associated with the pathogenesis of PCOS without HA. However, our study has limitations. First, the sample size is relatively small, and we cannot conclude definitively that there is an association between the markedly elevated plasma TMAO concentration and all PCOS patients without HA. In addition, we did not collect information on dietary intake, which is known to affect plasma TMAO concentrations. Third, TMAO is correlated with possibly altered gut microbiota, and whether this was an independent predictor (independent of other toxins) for the increase in inflammatory markers and for the phenotype in PCOS is unknown, as these were not analyzed in this study. Therefore, we could not determine whether these factors were associated with plasma TMAO concentrations in participants.

Since TMAO has multiple effects, including on thrombosis and platelet formation, inflammation, lipid metabolism, IR, glucose metabolism, cancer, and the development and progression of related diseases, and has been studied in CVD, it is expected to be a predictor for the early diagnosis, efficient evaluation and prognosis of the above diseases, and it may become a therapeutic target in related diseases. In this context, is TMAO associated with the pathogenesis of PCOS, or could its levels be a consequence of PCOS, similar to the manner in which TMAO levels are a consequence of increased weight? Further research on the role of TMAO may provide new ideas for the diagnosis, monitoring and clinical treatment of related diseases.

## Conclusions

Our study found that elevated plasma TMAO levels in PCOS without HA correlated with increased systemic inflammation but not IR directly. Elevated plasma TMAO levels may be associated with the pathogenesis of PCOS without HA and correlated with increased systemic inflammation. Further studies are needed to determine the suitability of TMAO as a predictive biomarker and to identify possible therapies for PCOS.

## Data Availability

In this study, the names and ID numbers of the patients were classified, according to Chinese law. Patients’ information can only be obtained from the hospital’s electronic medical record system on reasonable request if legal implications are fulfilled.

## Abbreviations

ALT: Alanine aminotransferase
AST: Aspartate aminotransferase
IFN-α: Interferon alpha
IFN-γ: Interferon gamma
IL-10: Interleukin 10
IL-12p70: Interleukin 12p70
IL-17A: Interleukin 17A
IL-18: Interleukin 18
IL-33: Interleukin 33
IL-6: Interleukin6
IL-8: Interleukin 8
MAPK: Mitogen-activated protein kinase
MCP-1: Monocyte chemo-attractant protein-1
PCOS: Polycystic ovary syndrome
TMAO: Trimethylamine-N-oxide
TNF-α: Tumor necrosis factor alpha
